# Impact of a Short Study Skills Course on Depressive Symptoms Among Medical Students

**DOI:** 10.7759/cureus.98874

**Published:** 2025-12-10

**Authors:** Eiad AlFaris, Abdullah M Ahmed, Farhana Irfan, Fahad D Alosaimi, Gominda Ponnamperuma, Haneen AL-Mazroua, Haytham AlSaif, Shaik Shaffi Ahamed, Lulu Alwazzan, Mohammed Akresh, Saud Al-hasani, Rakan A Aldoghmani, Mohammad Y Abdulghani, Faisal Jazzar, Abdullah Alzoghaibi

**Affiliations:** 1 KSU Chair for Medical Education Research and Development, King Saud University, Riyadh, SAU; 2 Department of Family and Community Medicine, College of Medicine, King Saud University, Riyadh, SAU; 3 Department of Psychiatry, College of Medicine, King Saud University, Riyadh, SAU; 4 Department of Medical Education, Faculty of Medicine, University of Colombo, Colombo, LKA; 5 Department of Pharmacology and Toxicology, College of Pharmacy, King Saud University, Riyadh, SAU; 6 Medical Education, Imam Mohammad ibn Saud Islamic University, Riyadh, SAU; 7 College of Medicine, King Saud University, Riyadh, SAU

**Keywords:** emotional well-being, impact, medical student, online course, study habit

## Abstract

Objectives

The study aimed to assess the impact of a study skills course on the prevalence and severity of depressive symptoms among third-year medical students.

Depression in this population can affect their education and future clinical practice, making it crucial to address and explore ways to mitigate the impact of studying in medical school on depressive symptoms and mental health.

Methods

An experimental cohort study following two groups prospectively was conducted between January and May 2022. The Study Skills Inventory (SSI) measured study skills, while the Patient Health Questionnaire (PHQ-9) assessed depressive symptoms in both the index and control groups, before and after the course. Of the 69 participants, 36 were in the index group, and 33 were in the control group. A six-session study skills course was conducted virtually via the Zoom (Zoom Communications, San Jose, CA, USA) platform due to restrictions imposed during the COVID-19 pandemic. The course incorporated a variety of teaching and learning strategies, including interactive lectures, small group discussions, educational materials, and skill-building exercises. In addition to descriptive statistics, mean ranks of the pre-post test scores of the index and control groups were compared using Mann-Whitney U and Wilcoxon Signed-Rank tests, as appropriate.

Results

Overall, session attendance was low. No statistically significant differences in depressive symptoms or study skills were detected between the two groups, either pre- or post-intervention.

Conclusion

This study concluded that the study skills course did not demonstrate a statistically significant reduction in depressive symptoms. Future research should evaluate similar interventions within more comprehensive curricula to assess their effects on psychological well-being.

## Introduction

Many health college students commonly experience mental health issues; depression being the most prevalent [1,2]. Depression is a common complex psychiatric disorder that deserves special attention, especially amongst medical students [3-6]. Two meta-analyses revealed the global pooled estimate of depression prevalence of (62,728) [5] and (132,068) [7] medical students as 28% and 48% respectively. The prevalence of depression among medical students varies by region, with the highest rates observed in the Middle East (43.6%), followed by North and South America (30.2%) and Asia (26.6%), while Europe has the lowest prevalence at 23.9% [4].

Additionally, research indicates that the average prevalence of depression among medical students in Saudi Arabia is 35.25% [8]. The adverse effects of depression on students' well-being can persist beyond graduation [9], potentially compromising patient care quality, safety, and professional conduct [3,10]. Depression also negatively impacts one's personal, social, and work life [11]. Hence, the emotional well-being of young future doctors impacts both patients and society [9,10,12]. Given this background, it is no surprise that students’ well-being is considered a major public health challenge and priority.

The validated scales most frequently used to screen for depression are Beck Depression Inventory II (BDI II) [13], Hamilton Rating Scale for Depression (HAM-D) [14], and Centre for Epidemiologic Studies Depression Scale (CES-D) [15]. Additionally, the Patient Health Questionnaire-9 (PHQ-9) has been recognized for its high reliability across diverse populations [16]. Among the many study skills scales, and based on a literature search, only a few validated inventories were found. For example, LASSI (Learning and Study Strategies Inventory) [17], ASSIST (Approaches and Study Skills Inventory for Students) [18], and DCSSI (Denver Congos Study Scale Inventory) tool [19], had several shortcomings, including being impractically long. The newly developed Study Skills Inventory (SSI) has demonstrated acceptable reliability, with a Cronbach’s alpha of 0.84, as well as supporting evidence for its validity [3].

A recent study found that depressive symptoms had a moderate, negative relationship with study skills and that fostering students' study skills contributed to their overall enjoyment of the educational experience [9]. In the broader context of mental health, exploration of study skills and their relation to depression is vital for devising preventive strategies [20]. Hence, understanding the impact of students' study skills and learning on students' mental illnesses, particularly depression, is crucial for health and education planners [21,22].

Students who employ ineffective study skills [23,24] may experience suboptimal academic performance and compromised emotional well-being [3,25,26]. A meta-analysis of 109 studies identified a significant relationship between psychosocial factors, study skills, and academic achievement [27]. For the purposes of this research, "study skills" are defined as the deliberate and strategic application of techniques for organizing and retaining new information, with the goal of enhancing learning efficiency [12,28,29].

It was hypothesized that the development of effective study skills, such as reading, concentration, time management, and emotional regulation-might enhance affective well-being and reduce depressive symptoms. The study aimed to assess the impact of a study skills course on the prevalence and severity of depressive symptoms among third-year medical students. The study aimed to assess the impact of a study skills course on the prevalence and severity of depressive symptoms among third-year medical students.

## Materials and methods

The study was conducted in the medical school of King Saud University (KSU) located in Riyadh, Kingdom of Saudi Arabia, from January 2022 to May 2022. The school offers a six-year baccalaureate degree program in medicine and operates on a segregated gender basis. The Institutional Review Board, College of Medicine, King Saud University, issued approval No. 21/0770/IRB, September 9, 2021. 

Study design

It is an experimental cohort study that followed two groups prospectively. The Index group attended a study skills course, whereas the control group was not exposed to the training course, and the dependent variable was Depressive symptoms severity score.

Target population

The target population was selected according to the inclusion/exclusion criteria in Table 1.

**Table 1:** Inclusion and exclusion criteria PHQ: Patient Health Questionnaire

Inclusion	Exclusion
Students who had mild, moderate, or moderately severe depressive symptoms based on the PHQ-9 instrument were used as the study population.	Students who displayed severe depressive symptoms as indicated by a PHQ-9 score between 20 and 27, were excluded from the study. This decision was made to prioritize their need for immediate medical care, before burdening them with an extra study skills course.
The second semester of the third year was selected to recruit participants as this is the time medical students have adequately accustomed themselves to undergraduate life and for logistic reasons as it is difficult or impossible to schedule course training sessions that accommodate all study years simultaneously.	Those with PHQ-9 scores 0-4 (Normal) were excluded because of the less likelihood of finding improvement in depressive symptoms.
	Students who had a previous enrolment to an academic study skills course (outside the school curriculum) as they have already received the index
	Students who were under treatment for a mental disorder.

Sample size

We hypothesize that the study skills index would improve the PHQ-9 score in the index group by 5 units. Based on a recent study among Saudi medical students and interns in different universities using the PHQ-9, it was found that the mean and the standard deviation were 12.1 and 7.17 units, respectively [30].

Using the sample size estimation formula for the two-sample Wilcoxon Mann-Whitney U-Test [31], with a two-sided α level of 0.05 and a power of 80%, a sample size of 33 subjects for each of the index and control groups was estimated, i.e., a total of not less than 66. The total target group for this study was 68 subjects.

Research instruments

The Study Skills Inventory (SSI) and Patient Health Questionnaire (PHQ-9) tools were administered for data collection.

*Study Skills Inventory (SSI)* 

The SSI comprises 23 statements categorized into five key areas: reading comprehension, concentration and memory, time management, emotional regulation, and general learning strategies. The total possible score for the SSI is 69.

Each statement is assessed using a four-point Likert scale, with response options as follows: 0 is Never (almost 0% of the time), 1 is Rarely (about 25% of the time), 2 is Usually (approximately 75% of the time), 3 is Always (nearly 100% of the time).

This tool has been previously validated and employed in a study involving medical students from the same institution, where it demonstrated high reliability and validity [3].

Patient Health Questionnaire (PHQ-9)

There are concerns that the identification of depressive symptoms may be dependent upon the scale that is employed for measurement [32]. Hence, the relationship between depressive symptoms and study skills may be scale-dependent. Addressing this consideration, the present study opted for the PHQ-9 scale to measure depressive symptoms, instead of the BDI-II, commonly used in many previous studies. The PHQ-9 is a brief, open-access questionnaire commonly utilized in research for depression screening. It serves as a self-administered assessment tool, comparable to other established depression rating scales. Comprising nine items, the PHQ-9 is more concise than many other depression measures while maintaining strong validity and reliability. It has undergone psychometric evaluation across 14 studies, making it one of the most extensively assessed depression screening tools [33]. The PHQ-9 is structured around the nine diagnostic criteria for depressive disorders as outlined in the DSM-IV (American Psychiatric Association, 1994) [34], and was validated as a screening instrument by Kroenke et al [16]. A recent systematic review reported its sensitivity and specificity for diagnosing major depressive disorder (MDD) to range from 37% to 98% and 42% to 99%, respectively [33]. Additionally, it exhibits good internal reliability, with Cronbach’s alpha values between 0.67 and 0.89 [33]. PHQ-9 scores fall within the range of 0 to 27, with each item rated from 0 (“not at all”) to 3 (“nearly every day”). The categories of depression severity are as follows: (0-4) None, (5-9) Mild, (10-14) Moderate, (15-19) Moderately Severe, (20-27) Severe.

Data collection method

Before the first session, both the index and control groups were required to complete the three instruments, namely the demographic questionnaire, the PHQ-9, and the SSI. This process was repeated after the final study skills session, three months after the pre-intervention assessment. Furthermore, those who did not fill out the three instruments post-intervention were reminded twice. The data collection was facilitated through a Google form during a Zoom (Zoom Communications, San Jose, CA, USA) meeting. The scores from both groups were labelled pre-test and post-test scores. To minimize potential bias and prevent contamination, students were provided with limited details regarding the study’s rationale. This measure was taken to reduce the risk of unintended information dissemination. Furthermore, students from the index group were specifically asked not to share the course material with their colleagues during the study period.

Sampling

The study sample was selected through a randomized process using Microsoft Excel (Microsoft Corp., Redmond, WA, USA) to generate random numbers within each sex category. Among the 219 eligible students, 82 individuals from the mild to moderate and moderately severe groups (scoring between 5 and 19) were randomly chosen and invited to participate. Only those who provided informed consent and confirmed their commitment to attending all required sessions, as instructed by the investigators, were randomly assigned to either the index or control group using Microsoft Excel. This was essential to ensure a consistent participation level throughout the study. The index group (36 students) was offered a comprehensive six-session study skill course spanning over two months. However, the control group (33 students) remained without intervention.

Study skills course

Due to restrictions imposed during the COVID-19 pandemic, in-person attendance was not feasible; therefore, all sessions were conducted virtually via the Zoom platform.

The course incorporated a variety of teaching and learning strategies, including interactive lectures, small group discussions, educational materials, and skill-building exercises. The course consisted of six sessions, each lasting between one and a half and two hours. Examples of the educational material used in the course are studygs.net [35] and otis.libguides.com [36].

In the first session, students completed the demographic questionnaire, the SSI, and the PHQ-9. Subsequently, a discussion of the following concepts through an engaging PowerPoint (Microsoft, Redmond, WA, USA) presentation and interactive study skills activities took place: (i) Exploration of their perception of the meanings of study, learn, and review. And analyzing students’ perceptions of the terms study, learn, and review; (ii) Emphasizing the significance of connecting new knowledge to prior learning. A handout was provided to the participants that covered the concepts in more detail.

In the second session, after exploring the learners’ practice, the focus was geared towards practical strategies for effective learning. Presentations on important aspects such as time management, memory, and concentration, with emphasis on basic study skills (e.g., grouping, chunking, interleaving, etc.); reading skills, and emotional management (study skills tips) were conducted. A handout for further reading was provided.

In the third session, the students completed the ‘activity paper and activity folder’ (explained in Appendix 1 and Appendix 2)**,** plus the videos given beforehand were discussed.

In the fourth session, the students were provided with brief reading material to comprehend and practice. Based on the study techniques used when studying the material, feedback was given.

The fifth session was in the form of students’ assignments and discussions.

The sixth session was in the form of discussing learning objectives and a recap of the course, with filling out the two questionnaires.

Building skills and self-efficacy were encouraged throughout the sessions. Examples of productive (or beneficial) study skill techniques include taking frequent breaks, not continuing to study the same topic for prolonged periods, revising often, using memory aids, enhancing understanding using diagrams (mind maps, flow charts, etc.), application of learning, Q & A sessions, etc. A separate list of useful or beneficial study techniques, based on the SSI, was developed and handed to the students. Furthermore, students were encouraged to openly express commitment to change their non-productive study skill techniques with colleagues and housemates.

Immediately following the study skills course, participants assigned to the training were invited to complete a satisfaction questionnaire to assess Kirkpatrick’s Level 1 (reaction) [37]. This questionnaire gathered feedback on various aspects, including content, instructional design, facilitation, and overall outcomes. Participants provided their responses using a four-point scale** **(Yes, to a high degree; Yes, to some degree; No; Not sure). No open-ended questions were included in the questionnaire [37]. 

Statistical analysis

Data analysis was conducted using the IBM SPSS Statistics for Windows, version 22 (IBM Corp., Armonk, NY, USA). Descriptive statistical analysis, such as frequency and percentages, was calculated. The mean ranks of the pre-test scores for the index and control groups were compared separately for the SSI and the PHQ-9 using the Mann-Whitney U test. The same procedure was then applied to compare the post-test scores of the index and control groups. The medians of the pre- and post-index groups for the SSI were compared using the Wilcoxon signed-rank test.

Finally, an evaluation of the effectiveness of the index was conducted through the comparison of pre- and post-index data and the students’ satisfaction level with study habits.

## Results

A total of 69 students participated in the trial, with male students comprising the majority (N = 39), 57%. All participants were single and resided with their families. The vast majority (N = 67), 97% were non-smokers (Table 2).

**Table 2:** Sociodemographic characteristics of the 69 students included in this trial

Characteristic		n (%)
Sex	Male	39 (56.5%)
	Female	30 (43.5%)
Marital status	Single	69 (100%)
	Married	0 (0 %)
Living with family	Yes	69 (100%)
	No	0 (0%)
Smoking	Yes	2 (2.9%)
	No	67 (97.1%)

Overall, session attendance was low, with an average attendance rate of (N = 15), 41%. More than half of the participants (N = 21), 58% attended only one or two sessions, while (N = 13), 36% attended three to four sessions. There is no statistically significant difference in the median ranks of total PHQ-9 before (p= 0.253) and after the course (p= 0.221) and SSI scores between the index and control groups, both before (p= 0.130) and after the course (p= 0.324) (Table 3).

**Table 3:** Comparison of total PHQ-9 and SSI scores between index and control groups using the Mann-Whitney U test SSI: Study Skills Inventory, PHQ-9: Patient Health Questionnaire

Outcome variable		Group	N	Mean rank	Z -value	U	p-value
Total (PHQ-9)	Before	Index	36	32.4	-1.143	500.0	0.253
		Control	33	37.9			
	After	Index	36	37.82	-1.224	492.5	0.221
		Control	33	31.92			
Total (SSI)	Before	Index	36	31.50	-1.516	468.0	0.130
		Control	33	38.82			
	After	Index	36	32.72	-.986	512.0	0.324
		Control	33	37.48			

Similarly, the paired ranks of PHQ-9 and SSI scores, for the index group both before and after the course, showed no statistically significant difference for the PHQ (p= 0.099) and the SSI course (p= 0.078) (Table 4).

**Table 4:** Comparison of PHQ-9 and SSI scores paired ranks among the index group using Wilcoxon signed-rank test SSI: Study Skills Inventory, PHQ-9: Patient Health Questionnaire

Index group		N	Mean rank	Z-value	p value
Total (PHQ-9)	Pre-test	36	(-ve) = 18.85 (+ve) = 16.20	-1.651	0.099
	Post-test				
Total (SSI)	Pre-test	36	(-ve) = 16.55 (+ve) = 17.23	-1.760	0.078
	Post-test				

Most students (N=28; 77%) assessed the course as good or excellent on the domains of teaching content, instructional design, facilitation, and goal achievement. Similarly, the majority (N=22; 61%) found that the course met their expectations, and around one third (N=12; 33%) claimed that it had somewhat met their expectations. Notably, there was a statistically significant improvement in the students’ satisfaction with their study habits post-intervention (p=0.006). A similar positive trend was observed in students’ perception of their study habits.

## Discussion

Our educational experimental study found no statistically significant improvement in mean depression scale PHQ-9 scores between the index and control groups following the study skills training. Likewise, no significant changes were observed in the mean PHQ-9 scores of the index group before and after the training. A study conducted among second-year medical students in Iran examined the impact of an optional learning and study skills course on students' learning strategies. The findings demonstrated a significant improvement in various learning strategy domains, including attitude, time management, information processing, study aids, and self-testing, with the index group showing higher scores (p < 0.05 for all). However, no significant improvement was observed in the motivational scale, a key factor linked to academic success [38]. Similarly, an accelerated prospective cohort study in Vietnam involving 623 medical students indicated that all self-regulated learning strategies subscales were significantly negatively correlated with depression scores, except for extrinsic goal orientation and peer learning [39]. Further multivariate analysis revealed that self-efficacy, help-seeking, time, and study environment were found to be significantly negatively associated with depression, even after adjusting for demographic and psychological covariates, including depression, anxiety, and stress [39].

Additionally, a pre-post assessment study evaluating the effectiveness of a cognitive-behavioral counselling service among 124 students (57% above the clinical threshold for distress) reported significant reductions in distress and symptom scores. The intervention also led to enhanced academic self-efficacy, with half of the participants demonstrating reliable positive changes [40].

Moir et al., in their extensive review, suggested that many skills beneficial for depression prevention and treatment were also essential competencies for medical practitioners [41]. These skills contribute to patient safety, quality of care, and overall professional development, supporting their integration into the medical curriculum. Ideally, wellness initiatives should be embedded within a holistic framework, aligning with institutional values and daily operations. A proactive, strength-based approach that encourages active participation from both students and faculty remains the most effective strategy for mitigating depression among medical students [41].

So, the question is why there was an absence of improvement in both the PHQ-9 (depression symptoms) score and the SSI study skills. This may be due to the poor attendance of students for the study skills course, online delivery of the study skills courses, the course being conducted during a stressful COVID-19 period, and the course being conducted parallel to other courses in the mainstream curriculum, leaving insufficient time for students to concentrate on the study skills course and their usual academic studies. Several factors probably contributed to the low attendance, such as time constraints, social and personal factors, lack of awareness of benefit or perceived irrelevance, and scheduling conflicts [42-44].

Furthermore, it is not difficult to imagine that the intervention in this study served as an additional challenge and burden to the students in the short term. This, in turn, might have hindered the anticipated improvement of depressive symptoms. The low attendance rate in the sessions meant that the intervention did not provide its best potential. More than half of the students (N=21; 58%) attended only one or two sessions. Additionally, the course sessions were delivered online, distance learning through Zoom, which may have allowed many students to attend only part of the sessions. We believe that low attendance is the most important drawback of this trial, as it is self-evident that one or two sessions on study skills are unlikely to have the optimal impact on depressive symptoms. When students were informally asked about the reason for their absence, many of them attributed this to the presence of other midterm exams, quizzes, or assignments. For positive change to take place, students need to go through phases of pre-contemplation, contemplation, and before acting and maintaining behavioural change [45].

If, however, the study skills learning course was conducted before the formal learning of the subject matter, the results may have been different. Based on these findings, we recommend that future research explore the effectiveness of offering the study skills course as either an optional or mandatory component in the early years of education, before formal learning sessions begin. However, it is important to note that the absence of improvement in both study skills and depressive symptoms within the index group does not necessarily imply that study skills have no impact on depressive symptoms. Instead, the lack of improvement in depressive symptoms may stem from the absence of a statistically significant enhancement in study skills. To improve study skills, the students may require more time to practice those skills than the present time offered. It is after such time only that an improvement in depressive symptoms can be expected. While satisfaction with the training program represents a fundamental yet preliminary level of evaluation, as outlined in Kirkpatrick’s evaluation model, it plays a crucial role in facilitating behavioral change [46]. Furthermore, there was a statistically significant improvement in students’ satisfaction with their study habits following the intervention. Although the satisfaction and perception results are promising, these findings are on the softer side of assessment rather than the more objective study skill scores.

Lessons learned

A meticulous design is paramount when planning any interventional study that involves medical students, and the study should guarantee an optimal uptake of the index. This may be achieved by either making the course mandatory or carrying out the study skills course before formal learning in the school.

Limitations and recommendations

The low attendance rate and the online format of the course may have introduced bias and significantly influenced the study’s findings. Low participation is a common challenge in educational research, particularly among medical students, who often have demanding and overloaded schedules. The low uptake is very likely to happen in human educational research, particularly among medical students, who often have demanding and overloaded schedules. Only third-year students were included because this is the time medical students have adequately accustomed themselves to undergraduate life, and for logistical reasons, as it is difficult or impossible to have course training sessions scheduled that fit the whole class (study years) simultaneously. Furthermore, it was found in a previous study that third-year medical students had the highest rates of depressive symptoms [1].

Despite efforts to prevent the control group from being exposed to the intervention materials, it remains uncertain to what extent students in the index group shared their learning with their peers. This potential knowledge transfer introduces a source of bias contamination, which may have affected the study’s scientific rigor. When designing interventional studies involving medical students, careful planning is essential to ensure optimal engagement with the intervention. One way to enhance participation is to integrate the study skills course into the curriculum as a mandatory component. Alternatively, offering the course before formal academic instruction begins could help maximize student involvement and the overall effectiveness of the intervention." For all the above reasons, it is obvious that the results cannot be generalized.

## Conclusions

Contrary to our initial hypothesis, this intervention study did not demonstrate a significant improvement in the depressive symptoms as indicated by the mean PHQ-9 mean score following a study skills course. Future research in this area should consider integrating such interventions as elective courses that contribute to students' grades or implementing study skills training before the commencement of the demanding formal curriculum to maximize its effectiveness

## Appendices

Appendix 1

**Table 5:** Session details

Session No.	Details
Session 1	Filling out the Demographic Questionnaire, Study Skills Inventory (SSI), and Beck Depression Inventory (BDI-II). An interactive discussion of the SSI results took place. Discussion of the following concepts through an engaging PowerPoint presentation and interactive study skills activities took place: exploration of their perception of the meanings of ‘study’, ‘learn’, and ‘review’. The importance of linking new learning to prior knowledge.
Session 2	After exploring the learners' practice, an interactive lesson discussing presentation on time management, memory, and concentration, with emphasis on chunking, etc., reading skills, and emotional management (study skills tips).
Session 3	Activity paper and activity folder. Plus, discussing videos given beforehand. A handout that covers the concepts in more detail was provided.
Session 4	Follow-up workshop (30-minute); to review the techniques previously introduced, followed by a discussion centered on the study techniques students had implemented and worked for them, as well as those who had struggled with them. At the end of the session, a feedback questionnaire with a question to denote whether they had attended the initial workshop or not. The students were provided with a short material to read, practice and given feedback.
Session 5	The fifth session was in the form of students’ assignments and discussions.
Session 6	The sixth session was in the form of working with Learning Objectives and a recap of the course with filling out the two questionnaires. This was followed by consolidation and summarization of what was learned.

Appendix 2

Session 3 Details

Students were given the activity packet with instructions not to open until asked and a blank 4×6 card. At the beginning of the session, a slide displaying eight 3- and 4-digit alphanumeric combinations (for memorization) was shown. Students were given two minutes and instructed to memorize the combinations without writing anything down.

Activity 1 (key and Riyal (local currency like penny in the USA) exercise): Each student was provided with a card and draw 2 circles. (Instruction was shown on the displayed slide via animations). It was explained that there are ten “pieces of information” on a Riyal, and students were asked to indicate how many pieces of information they were able to answer correctly, out of 10. The students were given two minutes to draw the front and back of a Riyal (from memory - no coins will be allowed).  At the end of the two minutes, the students were asked to score/grade their drawing.

The purpose of this activity for the students is to show how ineffective simply memorizing is, even if we just see the thing daily without understanding.

Approximately halfway through the session, Activity 2 was begun.

Activity 2 (for memorization, the alphanumeric combinations): Students were instructed to think briefly of the alphanumeric combinations presented at the start of the workshop and write them down (two minutes will be given to produce them). At the end of the two minutes, the students were asked to score/grade their written combinations. The purpose of this activity for the students is to know that memorization is better if made connections with previous schema or previous meaningful abbreviations.

During the session, the study techniques that were used before, during, and after the lesson were discussed with examples.  At the end of the session, students were given an anonymous feedback questionnaire to allow the students to immediately reflect on their individual lessons learned and a plan of action for the future study skills (intended techniques) to address any concerns in future sessions.
